# Factors associated with high heterogeneity of malaria at fine spatial scale in the Western Kenyan highlands

**DOI:** 10.1186/s12936-016-1362-y

**Published:** 2016-06-04

**Authors:** Amrish Y. Baidjoe, Jennifer Stevenson, Philip Knight, William Stone, Gillian Stresman, Victor Osoti, Euniah Makori, Chrispin Owaga, Wycliffe Odongo, Pauline China, Shehu Shagari, Simon Kariuki, Chris Drakeley, Jonathan Cox, Teun Bousema

**Affiliations:** Department of Medical Microbiology, Radboud University Medical Centre, Geert Grooteplein 26-28, 6525 GA Nijmegen, The Netherlands; European Programme for Public Health Microbiology Training, European Centre of Disease Prevention and Control (ECDC), Stockholm, Sweden; Department of Infectious and Tropical Diseases, London School of Hygiene and Tropical Medicine, Keppel Street, WC1E 7HT London, UK; Johns Hopkins Bloomberg School of Public Health, 615 North Wolfe Street, Baltimore, MD 21205 USA; Department of Mathematical Sciences, University of Bath, Claverton Down, Bath, BA2 7AY UK; Kenya Medical Research Institute, Mumias Road, Kisumu Station, Kisian, Kisumu, Kenya

**Keywords:** Malaria, *Plasmodium falciparum*, Hotspots, Heterogeneity, Transmission, Elimination, Risk-factors, Serology, Polymerase chain reaction

## Abstract

**Background:**

The East African highlands are fringe regions between stable and unstable malaria transmission. What factors contribute to the heterogeneity of malaria exposure on different spatial scales within larger foci has not been extensively studied. In a comprehensive, community-based cross-sectional survey an attempt was made to identify factors that drive the macro- and micro epidemiology of malaria in a fringe region using parasitological and serological outcomes.

**Methods:**

A large cross-sectional survey including 17,503 individuals was conducted across all age groups in a 100 km^2^ area in the Western Kenyan highlands of Rachuonyo South district. Households were geo-located and prevalence of malaria parasites and malaria-specific antibodies were determined by PCR and ELISA. Household and individual risk-factors were recorded. Geographical characteristics of the study area were digitally derived using high-resolution satellite images.

**Results:**

Malaria antibody prevalence strongly related to altitude (1350–1600 m, p < 0.001). A strong negative association with increasing altitude and PCR parasite prevalence was found. Parasite carriage was detected at all altitudes and in all age groups; 93.2 % (2481/2663) of malaria infections were apparently asymptomatic. Malaria parasite prevalence was associated with age, bed net use, house construction features, altitude and topographical wetness index. Antibody prevalence was associated with all these factors and distance to the nearest water body.

**Conclusion:**

Altitude was a major driver of malaria transmission in this study area, even across narrow altitude bands. The large proportion of asymptomatic parasite carriers at all altitudes and the age-dependent acquisition of malaria antibodies indicate stable malaria transmission; the strong correlation between current parasite carriage and serological markers of malaria exposure indicate temporal stability of spatially heterogeneous transmission.

## Background

Infectious disease transmission often displays heterogeneity of transmission in space and time [1]. Malaria forms no exception to this and in the last decade, considerable efforts have been made to improve estimates on the global and local burden of malaria transmission [2, 3, 4]. At a global scale, *Plasmodium falciparum* transmission is driven by temperature and aridity that limit the distribution and competence of *Anopheles* vectors [5]. However, at a micro-epidemiological scale in endemic areas, numerous factors influence malaria transmission dynamics including distance to the nearest mosquito breeding site [6–12] and house construction features [6, 8, 9, 13, 14]. Individual malaria risk may also be associated with human genetic factors [7, 8, 15] or with behavioural factors [6–8, 13] including those relating to occupation [16] and travel [17]. Variations in these factors over a small area can result in spatially heterogeneous transmission, resulting in foci or hotspots of malaria infection [2, 18]. Targeting hotspots may be a highly efficacious approach for malaria control [1, 19]; the operational feasibility of such targeted interventions depends on the stability of malaria hotspots in space and time [2, 20] and the ability to readily detect them.

In Africa, highland fringe areas have traditionally been associated with unstable malaria transmission, epidemics and unpredictable disease patterns [21, 22]. Over recent decades, however, it appears that this picture has been changing [23] with studies describing instances of relatively stable malaria transmission in the Kenyan highlands [24, 25], characterized by age-dependent acquisition of anti-malarial immunity [24, 26] and a substantial reservoir of asymptomatic malaria infections [27]. These studies were based on passively detected malaria cases [24, 25] and active surveillance in children [26, 27]; a more comprehensive, community-based, assessment of parasitological and serological outcomes in all age groups is needed to establish the macro- and micro-epidemiology of *P. falciparum* and to identify factors associated with exposure and infection so that more targeted and specific interventions can be locally deployed.

## Methods

### Study site and sampling

**Fig. 1:**
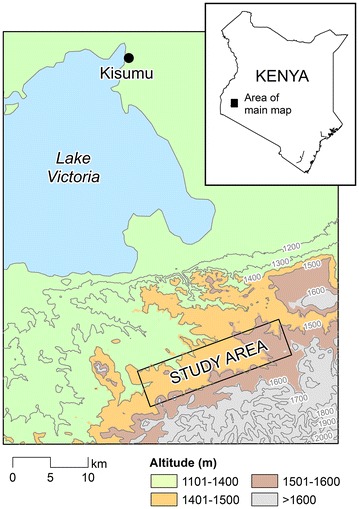
The study area in Western Kenya. The study area comprised a 5 × 20 km rectangle in Rachuonyo South District, Nyanza Province

This study was conducted in highland fringe localities (1350–1600 m altitude) in Rachuonyo South District, western Kenya (Fig. 1). The main malaria vectors in the area are *Anopheles funestus* and *Anopheles gambiae* sensu lato (*s.l.*) [28]. Malaria transmission is seasonal, with two peaks in malaria cases reflecting the bimodal rainfall pattern, with the heaviest rainfall typically occurring between March and June and a smaller peak between October and November each year. The study procedures have been described in detail elsewhere as part of an online clinical trial protocol [29]. Briefly, a 5 × 20 km (100 km^2^) area was selected and divided into 400 cells of 500 × 500 m that were further subdivided in four sub-cells of 250 × 250 m. All structures in the area were geo-located manually in ArcGIS [ArcGIS 9.2; Environmental Systems Research Institute, Redlands, CA, USA] using contemporaneous high-resolution satellite data [Quickbird; DigitalGlobe Services, Inc., Denver, CO, USA]. Where possible, a maximum of 16 compounds were chosen from each 500 × 500 m cell. The aim was to obtain measurements from ≥50 individuals per 500 × 500 m and sampling of individuals was guided by pre-defined age strata (≤5 years; 6–10 years; 11–15 years; 16–25 years and >25 years) to maximize the discriminative power of serological markers of exposure [30]. To ensure maximum geographical coverage, at least one compound was selected from each 250 × 250 m sub-cell while the number of compounds selected from each of the 250 × 250 m sub-cells was weighted by the density of structures in each sub-cell.

The survey was carried out in 2011, during what is considered to be the main malaria transmission season, between June and July. After initial consent during enumeration, participating compounds were visited and the name, gender, age, residency and travel history, use of insecticide-treated nets (ITNs), indoor residual spraying (IRS) in the past 12 months and sleeping times of each compound member were recorded. All compounds where at least one adult (>20 years) and one child (<15 years) were permanent residents (defined as sleeping regularly in the structure) qualified for enrolment during the survey. The axillary temperature of each compound member was measured by digital thermometer. For all febrile individuals a rapid diagnostic test [RDT; Paracheck^®^, Orchid BiomedicalSystems, Goa, India] detecting *P. falciparum*-specific histidine rich protein-2 was performed. A single finger prick sample was taken for haemoglobin (Hb) measurement using a HemoCue photometer [HemoCue 201+, Angelholm, Sweden] and three droplets of blood transferred onto a filter paper [3MM Whatman, Maidstone, UK] for serum and DNA [31]. All individuals with an Hb ≤11 g/dL were given haematenics; individuals with an Hb ≤6 g/dL were accompanied to a nearby health centre for further care. Febrile individuals who were parasitaemic by RDT were given artemether-lumefantrine [AL, Coartem^®^, Novartis, Switzerland]; women of child bearing age who were RDT positive were assessed for pregnancy and offered a pregnancy test if deemed appropriate. Febrile children below 6 months of age and pregnant women with malaria were referred to the nearest health facility for full assessment and treatment.

### PCR and serology

A combined extraction of DNA and elution of antibodies was performed on the samples collected, as described elsewhere [31]. Antibodies against *P. falciparum* apical membrane antigen 1 (AMA-1) and merozoite surface protein 1_19_ (MSP-1_19_) were measured in all samples by ELISA [32, 33]. Parasites were detected by nested PCR targeting the 18S rRNA gene [31, 34]. For logistical reasons, PCR was performed on a subset of all available samples (12,912/16,381).

### Geographical information

Altitude data for study compounds were derived from a high-resolution digital elevation model (DEM; ASTER GDEM). These DEM data were also used to derive a topographic wetness index (TWI) using the method of Cohen et al. [35]. Aggregated TWI estimates were derived for a 500 m circular window around each participating compound. Locations of rivers and streams were initially estimated using topographic modelling of DEM data and were later refined manually using Quickbird satellite data. The number of digitised structures within a 500 m circular window of each compound was used as a proxy for population density.

### Statistical analysis

Broad patterns of transmission intensity were described by fitting age-seroprevalence curves [32, 36] to samples collected from populations residing at different altitude bands 1350–1449 m, 1450–1499 m, 1500–1549 m and 1550–1641 m and quantifying parasite prevalence and antibody prevalence in 10 m altitude bands. For quantifying transmission intensity at a finer geographical scale, two individual level measures of transmission intensity were used: (1) combined antibody prevalence, i.e. seropositivity for AMA-1 and/or MSP-1_19_; and (2) PCR-detected parasite prevalence. Correlations between both metrics were determined using a Chi square test and hypothesis testing with significance determined where p < 0.05 and odds ratios (OR) and corresponding 95 % confidence intervals were calculated. Potential factors associated with antibody prevalence or parasite prevalence were explored using multivariate logistic regression models accounting for correlations between observations from the same compound. In these models, an equal correlation model (exchangeable) was used to specify the within-household correlation structure. All univariate analyses were adjusted for clustering of observations from the same household and age but no other factors. Adjustment for age was performed because this was a very important determinant of both parasite prevalence and antibody prevalence. For multivariate models a forward selection method was used, using a p value of 0.05 to retain variables in the model. All analyses were performed using Stata [v. 13, StataCorp].

### Ethical considerations

This study was approved by the ethical committees of the London School of Hygiene and Tropical Medicine (LSHTM 5721) and the Kenya Medical Research Institute (SSC 1802 & SSC2163). Approval was sought from district medical officers, local chiefs and communities. Individual informed consent was sought from all participants or guardians of those less than 18 years old by signature or a thumbprint accompanied with the signature of an independent witness. Assent was also sought from children above 13 years of age. As defined in the Kenya national guidelines, participants below 18 years of age who were pregnant, married, or a parent were considered “mature minors” and consented for themselves.

## Results

### Characteristics of the study population

In total 17,503 individuals were sampled, coming from 3213 compounds across a 100 km^2^ study area. The majority of individuals resided within a narrow altitude band of 1400–1550 m (92.5 %; 16,167/17,478). The median number of individuals per compound was 5 (interquartile range 3–7, range 1–29). As reported recently, PCR detected parasite prevalence was 21.2 % (738/3476) in children ≤5 years of age, 26.1 % (609/2337) in children aged 6–10 years, 24.8 % (462/1403) in children aged 11–15 years, 19.2 % (374/1574) in individuals aged 16–25 years and 14.6 % (480/3286) in individuals aged >25 years [37].

### Altitude and malaria risk

**Fig. 2:**
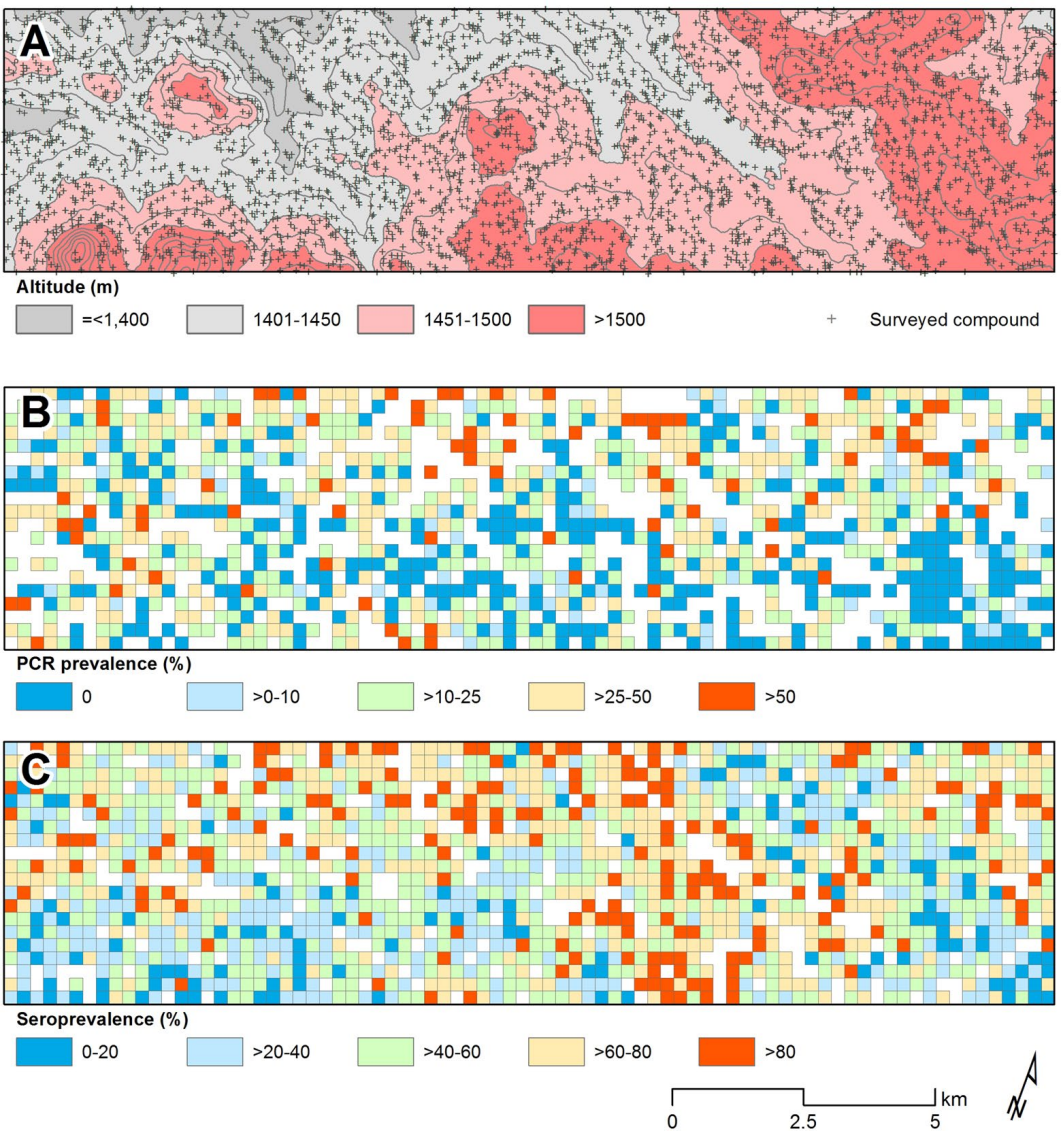
Maps representing altitude, spatial variation of nPCR and antibody prevalence in the study area Western Kenyan highlands Rachuonyo South District, Nyanza Province. **A** Detailed overview of the altitude in the study area. **B** Average nPCR prevalence in 250 × 250 m zones. **C** Average combined seroprevalence (for AMA-1 or MSP-1_19_) in 250 × 250 m zones

**Fig. 3:**
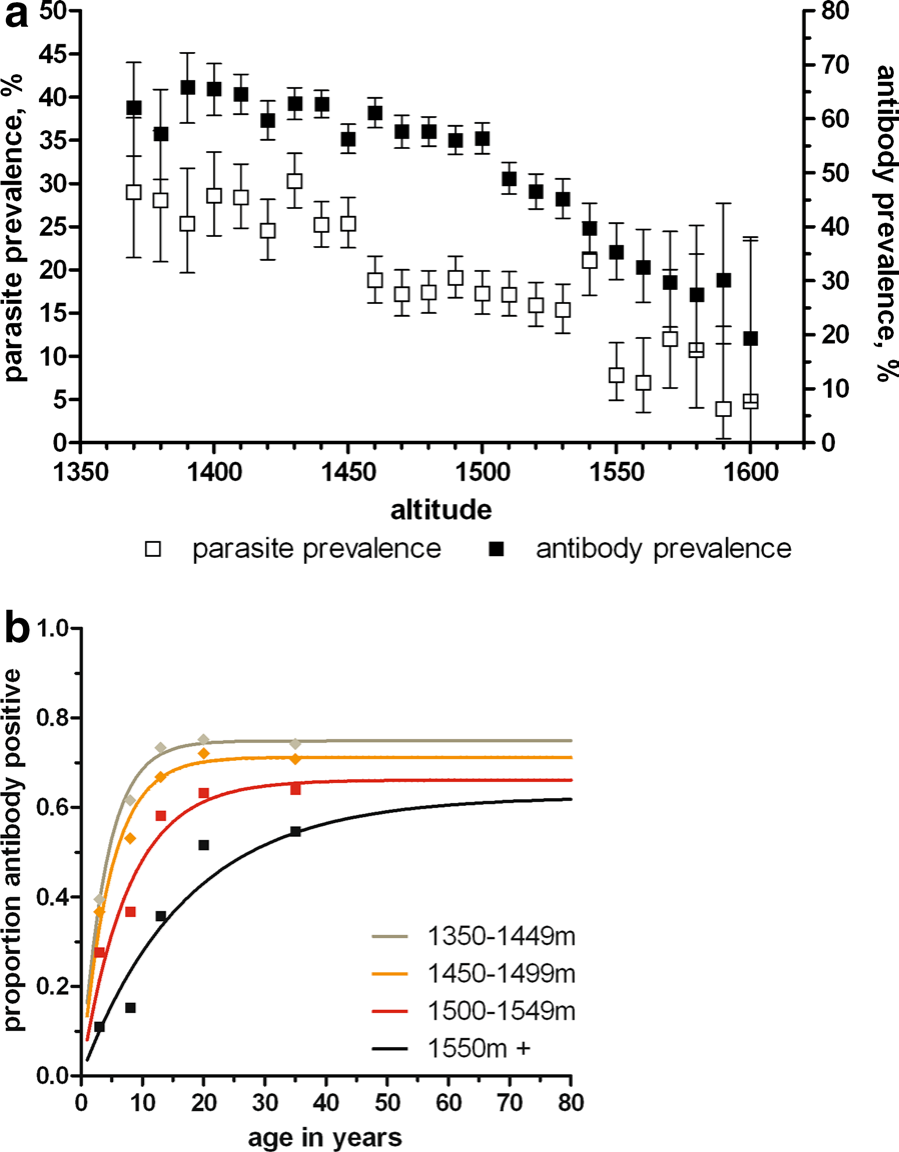
Parasite prevalence and antibody responses in relation to altitude. **a** Presents parasite prevalence by PCR (*open squares*) and malaria-specific antibody prevalence (MSP-1_19_ and/or AMA-1 antibodies detected by ELISA) showed a strong negative association with altitude (r = 0.92, p < 0.0001 for both associations). **b** Presents the age-dependent antibody acquisition at different altitudes. *Lines* show the fitted association between age and antibody positivity for individuals residing at 1350–1449 m (*grey line*, n = 4967), 1450–1499 m (*orange line*, n = 6252), 1500–1549 m (*red line*, n = 4361) and 1550 meters and above (*black line*, n = 777). *Symbols* indicate parasite prevalence estimates for children below 5 years of age, 6–10 years, 11–15 years, 16–25 years and >26 years. Symbols are plotted at the median age for the different categories; for the highest age category parasite prevalence is plotted at 35 years

The proportions of individuals with fever, clinical malaria (a positive RDT in combination with temperature ≥37.5 °C), parasites detected by PCR, malaria-specific antibody responses (Fig. 2) and anaemia (Hb <11 g/gL) were all negatively associated with increasing altitude (Table 1; p < 0.001 for all comparisons). The median age of individuals diagnosed with clinical malaria was 6 years (interquartile range 3–10 years), although 12.5 % (37/295) individuals with clinical malaria were >15 years of age (range 0–46 years); 93.2 % (2481/2663) of PCR-detected malaria infections were sub-clinical. The mean age of individuals with clinical malaria was not associated with altitude (p = 0.40). When altitude was categorized in bins of 10 m, there was a strong negative association between altitude and PCR parasite prevalence (Fig. 3a; r = 0.92, p < 0.0001) and malaria antibody prevalence (r = 0.92, p < 0.0001). Age-seroprevalence curves were fitted for antibody responses to MSP-1_19_ and/or AMA-1 for the four altitude bands and indicated a clear gradual decline of the seroconversion rate with increasing altitude (Fig. 3b). There are no apparent observed ‘steps’ in age-seroprevalence curves that can indicate changes in exposure due to intervention and/or age-associated behavioral changes patterns such as travel to malaria endemic regions outside the area of residence [38].

**Table 1:** Characteristics of participants in the cross-sectional survey

	1350–1449 m	1450–1499 m	1500–1549 m	1550–1650 m	Total
Number of participants (number of compounds)	5424 (1108)	6363 (1235)	4579 (816)	839 (140)	17,503 (3213)
Age, % (n/N) (years)					
<5	26.4 (1429/5424)	27.0 (1791/6636)	27.1 (1239/4579)	27.7 (232/839)	26.9 (4701/17,503)
6–10	18.9 (1024/5424)	18.1 (1202/6636)	18.0 (822/4579)	19.4 (163/839)	18.4 (3215/17,503)
11–15	15.2 (823/5424)	13.9 (923/6636)	15.0 (685/4579)	14.3 (120/839)	14.6 (2552/17,503)
16–25	13.7 (744/5424)	15.3 (1018/6636)	14.8 (677/4579)	16.5 (138/839)	14.7 (2579/17,503)
>26	25.9 (1404/5424)	25.7 (1702/6636)	25.3 (1156/4579)	22.2 (186/839)	25.5 (4456/17,503)
Fever, % temperature > 37.5 °C, % (n/N)	4.0 (216/5423)	3.1 (204/6631)	2.5 (116/4575)	1.3 (11/839)	3.1 (547/17,468)
Clinical malaria, % (n/N)^a^	2.3 (126/5423)	1.8 (120/6631)	1.1 (48/4575)	0.12 (1/839)	1.7 (295/17,468)
Parasite prevalence, % PCR positive (n/N)	27.2 (1111/4083)	19.6 (900/4599)	16.7 (592/3548)	8.1 (54/664)	20.6 (2663/12,912)
Antibody prevalence, % positive (n/N)^b^	62.6 (3108/4967)	58.0 (3623/6252)	48.3 (2104/4361)	31.7 (246/777)	55.5 (9092/16,381)
Anaemia, % (n/N)					
Severe (<6 g/dL)	0.6 (30/5282)	0.7 (46/6391)	0.4 (19/4366)	0.2 (2/818)	0.6 (97/16,878)
Moderate (<8 g/dL)	3.1 (163/5282)	3.4 (214/6391)	2.6 (112/4336)	2.0 (16/818)	3.0 (505/16,878)
Mild (<11 g/dL)	26.0 (1374/5282)	23.0 (1472/6391)	21.5 (938/4336)	20.2 (165/818)	23.4 (3954/16,878)

^a^Clinical malaria is defined as fever with a positive RDT with measured temperature > 37.5 °C

^b^Prevalence of antibodies against *P. falciparum* MSP-1_19_ and/or AMA-1

### Micro-epidemiological patterns in malaria transmission intensity

There was considerable inter-compound variation in antibody prevalence and PCR parasite prevalence. In some compounds no members were antibody positive (6.6 % of all compounds with ≥three sampled inhabitants, 178/2709) or PCR parasite positive (49.6 %, 1239/2496) while for other compounds ≥80 % of all compound members were antibody (24.1 %, 652/2709) or parasite positive (5.1 %, 126/2496). Malaria antibody prevalence and parasite prevalence were strongly correlated at an individual level (OR 1.94, 95 % confidence interval 1.77–2.13, p < 0.001) [37]. This association was apparent for all age groups but was strongest for children below 6–10 years of age (OR 3.16, 95 % CI 2.58–3.86, p < 0.001) and weakest for adults above 25 years of age (OR 1.45, 95 % CI 1.15–1.83, p = 0.002).

In univariate analysis, individual-level parasite prevalence and antibody prevalence was associated with age. When adjusting for age, the following factors were associated with parasite prevalence and antibody prevalence: the presence of eaves in the sleeping room, reported bed net use, construction of the sleeping room walls with mud, distance to water, altitude, the proportion of parasite positive individuals within a 500 m radius of the compound and the proportion of malaria antibody positive individuals within a 500 m radius of the compound (Table 2). In a multivariate model that was constructed using forward selection of variables, nPCR parasite prevalence was negatively associated with bed net use, altitude and maximum TWI, and positively associated with mud walls, the presence of open eaves (Table 2). Similarly, malaria antibody prevalence was negatively associated with bed net use, distance to water and altitude, and positively associated with the presence of open eaves and minimum TWI (Table 2). Reported travelling in the previous 3 months was not associated with individual-level malaria risk. At all altitudes, including the highest altitude band with the lowest level of infection [8.1 % (54/664)], there were compounds where ≥80 % of inhabitants were parasite positive. The proportion of compounds with parasite prevalence ≥80 % was 7.6 % (68/896) at 1350–1449 m, 5.4 % (56/1041) at 1450–1499 m, 4.8 % (35/733) at 1500–1549 m and 2.3 % (3/128) at >1550 m (test for trend, p = 0.002). High-risk compounds were characterized by mud walls of sleeping structures (OR 1.94, 95 % CI 1.00–3.79, p = 0.05), a higher PCR parasite prevalence in the surrounding community (OR 1.07, 95 % CI 1.05–1.09, p < 0.001) and a higher minimum TWI (OR 2.60, 95 % CI 1.37–4.95, p = 0.004).

**Table 2:** Factors associated with malaria parasite prevalence or antibody prevalence

	Parasite prevalence		Antibody prevalence	
	OR^a^	Adjusted OR (95 % CI)^a^	OR^a^	Adjusted OR (95 % CI)^a^
Individual characteristics				
Age ( years)				
≤5	1 (reference)	1 (reference)	1 (reference)	1 (reference)
6–10	1.27 (1.13–1.42)	1.26 (1.10–1.46)	1.88 (1.71–2.06)	2.35 (2.10–2.63)
11–15	1.22 (1.07–1.39)	1.25 (1.08–1.47)	3.51 (3.17–3.89)	4.70 (4.15–5.33)
16–25	0.92 (0.81–1.04)	0.89 (0.76–1.04)	4.57 (4.13–5.07)	6.57 (5.79–7.46)
≥26	0.64 (0.57–0.73)	0.61 (0.53–0.71)	4.33 (3.97–4.72)	6.05 (5.43–6.73)
Bed net use	0.79 (0.71–0.88)	0.82 (0.72–0.93)	0.85 (0.79–0.93)	0.89 (0.81–0.98)
House structure				
Mud wall	1.46 (1.25–1.71)	1.26 (1.05–1.50)	1.14 (1.03–1.27)	
Open eaves	1.25 (1.10–1.41)	1.16 (1.01–1.34)	1.28 (1.17–1.39)	1.33 (1.21–1.46)
Environment				
Distance to water				
<250 m	1 (reference)		1 (reference)	1 (reference)
250–500 m	1.04 (0.89–1.22)		0.97 (0.87–1.09)	1.04 (0.92–1.19)
500–999 m	0.83 (0.71–0.96)		0.66 (0.59–0.74)	0.91 (0.80–1.03)
1000 m+	0.78 (0.62–0.98)		0.44 (0.38–0.52)	0.81 (0.67–0.97)
Altitude (m)				
1350–1449	1 (reference)	1 (reference)	1 (reference)	1 (reference)
1450–1499	0.63 (0.55–0.72)	0.84 (0.72–0.98)	0.83 (0.75–0.91)	0.90 (0.81–1.01)
1500–1549	0.52 (0.45–0.61)	0.68 (0.56–0.81)	0.57 (0.51–0.64)	0.79 (0.69–0.89)
1550–1650	0.25 (0.17–0.37)	0.43 (0.28–0.66)	0.28 (0.23–0.35)	0.71 (0.56–0.90)
Parasite prevalence 500 m radius	1.06 (1.05–1.06)	1.07 (1.06–1.07)	1.03 (1.02–1.03)	
Antibody prevalence 500 m radius	1.02 (1.02–1.03)		1.04 (1.03–1.04)	1.04 (1.03–1.04)
TWI (minimum)			1.91 (1.70–2.16)	1.23 (1.06–1.41)
TWI (mean)			2.16 (1.94–2.41)	
TWI (maximum)	1.03 (1.01–1.05)	0.97 (0.95–1.00)	1.09 (1.07–1.10)	

^a^Adjusted for clustering on compound level. The odds ratio and 95 % confidence interval of household and geographical factors in relation to antibody prevalence, after adjustment for age and clustering of observations; TWI is topographical wetness index

## Discussion

This study, conducted in the Kenyan highlands, shows the occurrence of ongoing stable malaria transmission across an altitudinal range of 1350–1600 m, characterized by marked spatial heterogeneity. The age-dependent acquisition of malaria antibodies, strong correlation antibody prevalence and current parasite prevalence, along with the considerable asymptomatic reservoir of *P. falciparum* infections in all age groups, suggests that malaria transmission is relatively stable in the study setting.

Much of the highlands of East Africa represent fringe regions between stable and unstable malaria transmission; seasonal and spatial patterns in malaria transmission are affected to some degree by annual variations in rainfall but primarily by ambient temperature [39]. The notion that malaria is largely absent in areas higher than 1500 m [40] has been challenged by findings of a large asymptomatic reservoir of malaria infections at altitudes [27] and an age-dependent acquisition of clinical immunity to malaria infections in highland communities [24]. In this study area area at 1350–1600 m above sea level, the special epidemiology of malaria infections was determined and markedly heterogeneous malaria transmission was observed. This is commonly observed in areas of low endemicity [2, 20, 25] and heterogeneity in clinical malaria cases has previously been reported in the Kenyan highlands [25]. This study adds detail to previous findings by describing the fine-scale spatial distribution of asymptomatic parasite carriage and immunological evidence of previous malaria exposure in a highland area. PCR-detectable *P. falciparum* infections were very common in this highland setting, apparently asymptomatic and negatively correlated with altitude. Parasite prevalence by PCR was 27.2 % in the population residing at 1350–1449 m, 19.6 % at 1450–1499 m, 16.7 % at 1450–1549 m and 8.1 % at 1550–1650 m. Using an equation model fitted to 86 surveys that determined parasite prevalence by microscopy and PCR [41], the corresponding parasite prevalence could be estimated by microscopy at 9.7 % (95 % CI 8.3–11.2) at 1350–1449 m, 6.1 % (95 % CI 5.1–7.3) at 1450–1499 m, 5.0 % (95 % CI 4.1–6.1) at 1450–1549 m and 2.0 % (95 % CI 1.3–3.0 %) at 1550–1650 m. Only a small fraction of these *P. falciparum* infections resulted in fever at the time of sampling and apparently asymptomatic parasite carriage was prevalent at all altitudes [27] and in all age-groups [41]. Recently, a manuscript summarized the evidence on the clinical consequences of chronic low density infections, arguing that many infections are incorrectly classified as asymptomatic and have considerable health consequences in terms of anaemia, chronic inflammation, school performance and bacterial infections [42]. Since the cross-sectional design of this study does not allow to determine whether the nPCR detected infections had clinical implications for the study population, it cannot be concluded with certainty whether the detected infections were indeed asymptomatic. However, concurrent clinical symptoms were reported by a small minority of the examined population and infections were probably chronic in nature. Although individuals with limited previous exposure may harbor low density infections [41], the high prevalence of apparently asymptomatic parasite carriage, absence of travelling as obvious risk factor for malaria and the gradual age- and altitude-dependent acquisition of antibody responses to *P. falciparum* antigens at different altitudes suggests stable local malaria transmission in the area.

Whilst travel, a known risk factor for malaria in highland areas [43, 44], was not significantly associated with individual risk of malaria infection or antibodies, several household factors such as the presence of open eaves and mud walls [6, 9] were statistically significant predictors of malaria risk. This suggests that relatively simple household improvements may decrease malaria risk [45, 46] in a region where the perceived and measured indoor exposure to malaria vectors is low [28]. Altitude, distance to water and the proportion of antibody or parasite positive individuals in the immediate vicinity of a compound were statistically significant and biologically plausible factors associated with malaria risk. The latter could suggest considerable between household transmission.

## Conclusions

Evidence of relatively stable malaria transmission in this site at 1350–1600 m altitude was observed. Altitude was a major driver of malaria transmission in the study area, even across narrow altitude bands. Although malaria risk was spatially heterogeneous, the strong correlation between current parasite carriage and serological markers of malaria exposure and other established risk factors for malaria indicate temporal stability of geographical patterns in malaria exposure. The fine scale heterogeneity in this low-endemic setting may reflect a likely scenario for more endemic areas with active and effective control programmes as they reduce transmission to increasingly low levels. A priori knowledge of the factors that influence residual malaria in foci of low transmission is likely to further expedite control and elimination attempts.
